# Life cycle impact assessment of biofuels derived from sweet sorghum in the U.S.

**DOI:** 10.1186/s13068-021-02009-6

**Published:** 2021-08-05

**Authors:** Karla G. Morrissey, Greg Thoma, Dora E. López

**Affiliations:** 1 Department of Chemical Engineering, 1 University of Arkansas, Fayetteville, AR 72701 USA; 2 Sarkeys Energy Center, Melbourne School of Petroleum and Geological Engineering, 100 E Boyd, SEC-1210, Norman, OK 73019-1003 USA

**Keywords:** Sweet sorghum, Fermentation, Thermochemical conversion, Pyrolysis, Hydroprocessing, Renewable diesel, Renewable gasoline, Bio-oil

## Abstract

**Background:**

The objective of this study was to evaluate the environmental impact of the production of a range of liquid biofuels produced from the combination of fermenting sorghum stalk juice (bioethanol) and the pyrolysis/hydrotreatment of residual bagasse (renewable gasoline and diesel). Life cycle impact assessment (LCIA) was performed on a farm-to-wheels system that included: (i) sorghum farming, (ii) juice extraction, (iii) juice fermenting, (iv) bagasse pretreatment, (v) bagasse thermochemical treatment (pyrolysis, hydroprocessing, and steam reforming), and (vi) typical passenger vehicle operation. LCIA results were compared to those of petroleum fuels providing the equivalent functional unit—cumulative kilometers driven by spark ignition direct injection (SIDI) vehicles utilizing either renewable gasoline or ‘bioE85—a blend of bioethanol and renewable gasoline,’ and a compression ignition direct injection (CIDI) vehicle utilizing renewable diesel produced from 76 tons of harvested sweet sorghum (1 ha).

**Results:**

Sweet sorghum biofuels resulted in a 48% reduction climate change impact and a 52% reduction in fossil fuel depletion. Additionally, reduced impacts in ozone depletion and eutrophication were found (67% and 47%, respectively). Petroleum fuels had lower impacts for the categories of non-carcinogenic health impact, smog, respiratory effects, and ecotoxicity, showing tradeoffs between sorghum and petroleum fuels.

**Conclusion:**

Overall, sorghum biofuels provide advantages in environmental impact categories including global warming potential, fossil fuel depletion and eutrophication, showing potential for sorghum as a promising second-generation feedstock for fuel.

**Supplementary Information:**

The online version contains supplementary material available at 10.1186/s13068-021-02009-6.

## Background

There exist ongoing efforts to sustainably meet the world’s ever-increasing energy demands. Fuels produced from second-generation feedstocks such as agricultural crop residues have shown to be promising as they are not an immediate food source [11, 12]. As it is a second-generation feedstock, sweet sorghum has been determined to be promising as a fuel source. In comparison to corn ethanol and sugarcane ethanol, sweet sorghum is advantageous in that it has: (1) ample sugar content in its stalk that can be directly fermented (16–18% fermentable sugars) [33]; (2) low water and fertilizer requirements for farming (only 1/3 the amount of water per hectare compared to sugarcane) [5]; (3) higher drought and salt resistance compared to other agricultural crops and overall adaptability to a variety of climates [18, 33], and (4) a harvesting season that allows for double cropping with sugar farming [18, 22]. Additionally, sweet sorghum has superior residue/crop ratios (1:3) and comparable estimated ethanol yields of approximately 0.3 L bioethanol per kg of dry biomass in comparison to other second-generation feedstocks such as corn stover, barley and wheat straw [11].

The advantages of sweet sorghum as a second-generation biofuel feedstock has sparked research on the various genotypes of this crop and their fermentable sugar content [24, 28, 33], sugar extraction methods and fuel conversion pathways [21, 22], and the economic and environmental feasibility of various biofuel processing pathways from either its grain, stem juice, or bagasse [6, 9, 21, 30].). Ethanol production methods that have been developed for sorghum and other cellulosic feedstocks include biochemical and thermochemical conversion pathways. Previous life cycle assessment (LCA) studies that focus on sweet sorghum fuels have evaluated primarily biochemical pathways that deal with grain-based or sugar-based feedstock [4, 17, 27, 39] and residual bagasse conversion into cellulosic ethanol via batch, continuous or advanced solid-state fermentation [9, 10, 37]. Table 1 summarizes the part of sweet sorghum used for biofuel production, method used for biofuel conversion, co-products and their utilization, and functional unit used for the evaluation.

**Table 1:** Summary of LCA studies of sweet sorghum biofuels

LCA study	Sorghum feedstock	Biofuel conversion pathway	Co-products	Bagasse handling	Functional unit	Reported reduction in GHG emissions^d^
[39]	Grain, stalk juice^a,b^	Fermentation	Bagasse	Returned to field	1 L of bioethanol produced	52 to 69%
[10]	Grain, stalk juice, bagasse^a−c^	Fermentation	Distillers grains, grain, vinasse	Feedstock for CHP, cellulosic ethanol	1 MJ of bioethanol	35 to 72%
[9]	Stalk juice^b^	Fermentation	Bagasse	Heat source, electricity, animal feed, cellulosic ethanol	1 L of bioethanol produced	–
[37]	Sorghum stem^b^	Continuous solid-state fermentation	Grain, vinasse	-	1000 L of bioethanol produced	–
[17]	Sorghum stem^b^	Advanced solid-state fermentation	Grain, bagasse	Steam generation, forage	1 MJ of bioethanol	36.7 to 48%
Aguilar-Sánchez et al. 2018	Sorghum stem^b^	Fermentation	Bagasse	Waste, CHP	Tons sorghum biomass harvested and harnessed from 1 ha of land in 1 year	− 20 to 200%
[27]	Sorghum stem^b^	Advanced solid-state fermentation	Distillers grains, grain	Steam generation, fertilizer	1 MJ of bioethanol	20 to 66%

^a^Grain sorghum

^b^Sweet sorghum

^c^Forage sorghum

^d^Compared to gasoline on functional unit given and may represent range of results from various scenarios used in each study

All of these LCA studies evaluate the biochemical pathways to produce biofuels from various types of sorghum, with some utilizing bagasse as a feedstock for heat or electricity. No studies were found in the literature that also investigate the environmental impacts of co-producing sorghum bagasse liquid biofuels. Some studies in the literature have investigated co-production of sugar-based biofuels and lignocellulosic biofuels in the same biorefinery, including biofuels from wheat straw [36] and macroalgae [26]. In co-producing bioethanol and bio-oil from wheat straw, biofuel mass and energy yields nearly doubled compared to biofuels produced solely from bioethanol production [36]. Additionally, while biochemical production of sweet sorghum biofuel has been shown in these studies to have significant GHG emission reductions compared to conventional fuel (some show greater than 50% reductions in global warming potential, qualifying sorghum as an “advanced biofuel”, [19]), biochemical conversion of lignocellulosic feedstocks still faces challenges of low efficiencies and high processing and enzyme costs [1, 13, 16].

Alternative pathways for cellulosic feedstocks have been researched and developed in recent years to produce alcohols and fuels from thermochemical pathways including gasification, pyrolysis, and hydrothermal liquefaction [11]. Previous studies have investigated the cost-effectiveness of these thermochemically produced biofuels in the last decade [6, 14, 25, 35, 41], and some analyses have shown that thermochemical pathways of fast pyrolysis and hydrothermal liquefaction of biomass have the potential to be more cost-competitive compared to biochemical methods [8, 30, 40].

Fast-pyrolysis is a direct liquefaction method in which biomass is heated in an oxygen-free environment with high heat transfer rates; high energy transfer to biomass particles breaks down macro-compounds including cellulose, hemicellulose, and lignin to produce condensible liquid (bio-oil), non-condensible gases (syngas) and charcoal (biochar) [34]. The resulting condensible liquid is then upgraded via catalytic hydrotreatment to naphtha-range and diesel-range fuels [2, 3, 25]. While these pathways may be more economical than others, the goal of producing biofuels is to provide a fuel with reduced environmental burdens compared to those of petroleum fuels. Therefore, the environmental profile of any potential biofuel production process must be quantified and compared to that of conventional fuels. Currently, there are no LCAs available in the literature that report on the environmental impacts of a sweet sorghum integrated biorefinery that applies fast pyrolysis to residual bagasse followed by hydrotreating of the resulting bio-oil to expand the suite of biofuel products from sweet sorghum. However, the need for such a study has recently been identified in the literature [35]. Expanding the number of products available from sweet sorghum and other types of biomass using thermochemical pathways may further reduce GHG emissions and help meet energy demands [25]. The objective of this study was to conduct an LCA of sweet sorghum biofuels—bioethanol, renewable gasoline and renewable diesel—derived from all parts of the sorghum plant.

## Results and discussion

### Sorghum biofuel yields

We assumed a yield of 76 t of harvested sorghum per hectare [10], total yields of bioethanol, renewable gasoline and renewable diesel were estimated and used as reference flows to fulfill the functional unit for the study. Production of 5122 L, 2705 L and 834 L (4041 kg, 1947 kg, and 649 kg, respectively) were determined for bioethanol, renewable gasoline, and renewable diesel, respectively. A bioE85 fuel blended from bioethanol and renewable gasoline, and renewable gasoline were modeled as end-use products to be combusted in a SIDI passenger vehicle modeled using The Greenhouse gases, Regulated Emissions, and Energy use in Technologies (GREET) model 2020. Renewable diesel was assumed to power a CIDI vehicle. The mass balance and schematic of energy flows are provided as Additional file 1. Based on each fuel’s energy content and vehicle energy requirements adopted from GREET, a maximum travel distance of 58,488 km, 20,252 km, and 11,250 km were found for bioE85, renewable gasoline, and renewable diesel, respectively [38].

### Basis for LCIA comparison of biofuels vs. petroleum fuels

The basis for comparison used to evaluate environmental emissions was the cumulative vehicle km (v-km) achievable from sorghum biofuels originating from sweet sorghum stalk juice and bagasse. The reference flows (liters of fuel) of petroleum fuels required to deliver the same distance driven were calculated based on this cumulative v-km found for sorghum biofuels.

### Life cycle impact assessment results

**Fig. 1:**
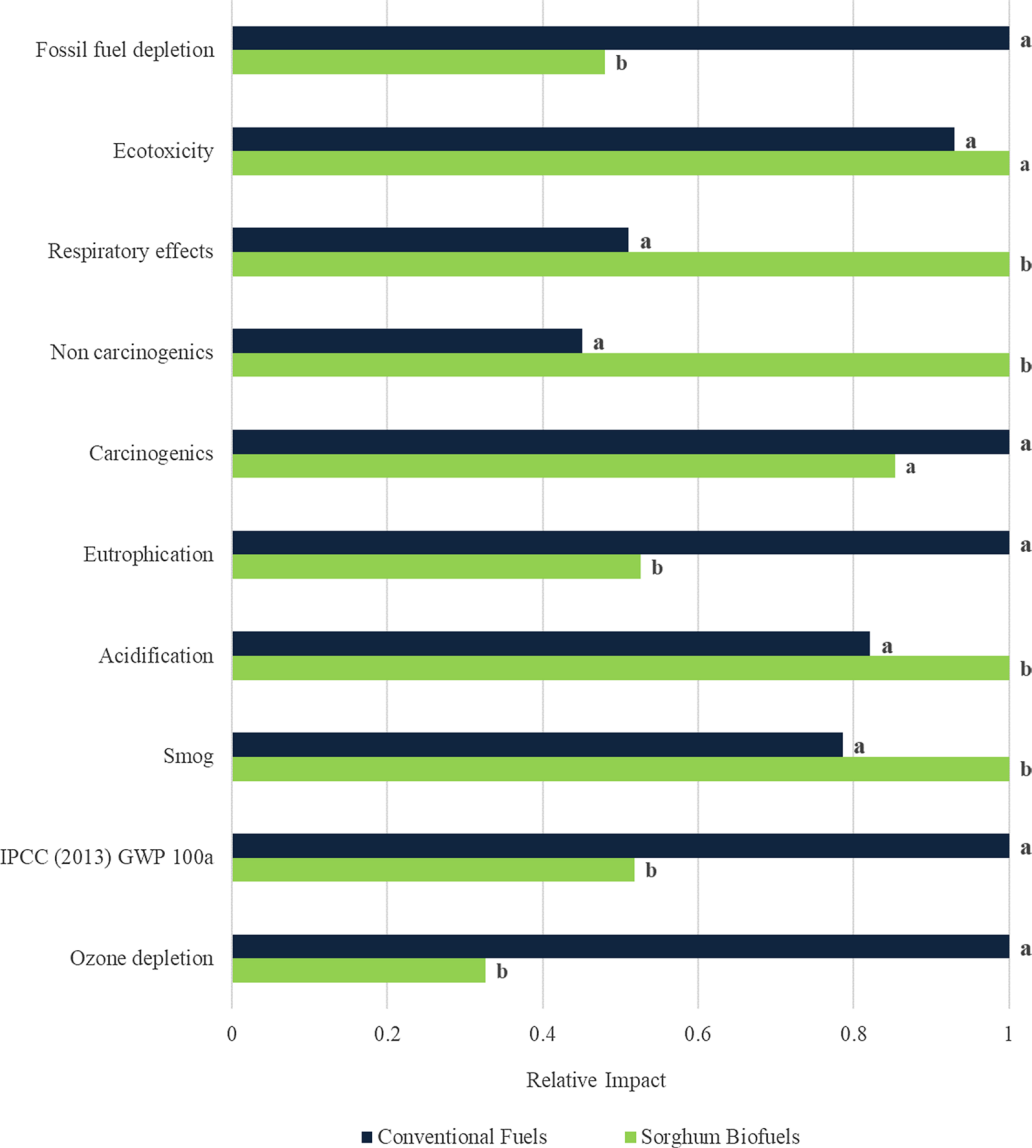
Environmental impact comparison of sorghum biofuels and petroleum fuels. Bars with different letters for each impact category are statistically different (*p* < 0.0001)

Figure 1 provides the comparison of the sweet sorghum-derived fuels and petroleum fuels for different categories: fossil fuel depletion, ecotoxicity, respiratory effects, carcinogenics, eutrophication, acidification, smog, IPCC (2013) Global Warming Potential 100 years (GWP 100a), and ozone depletion.

**Fig. 2:**
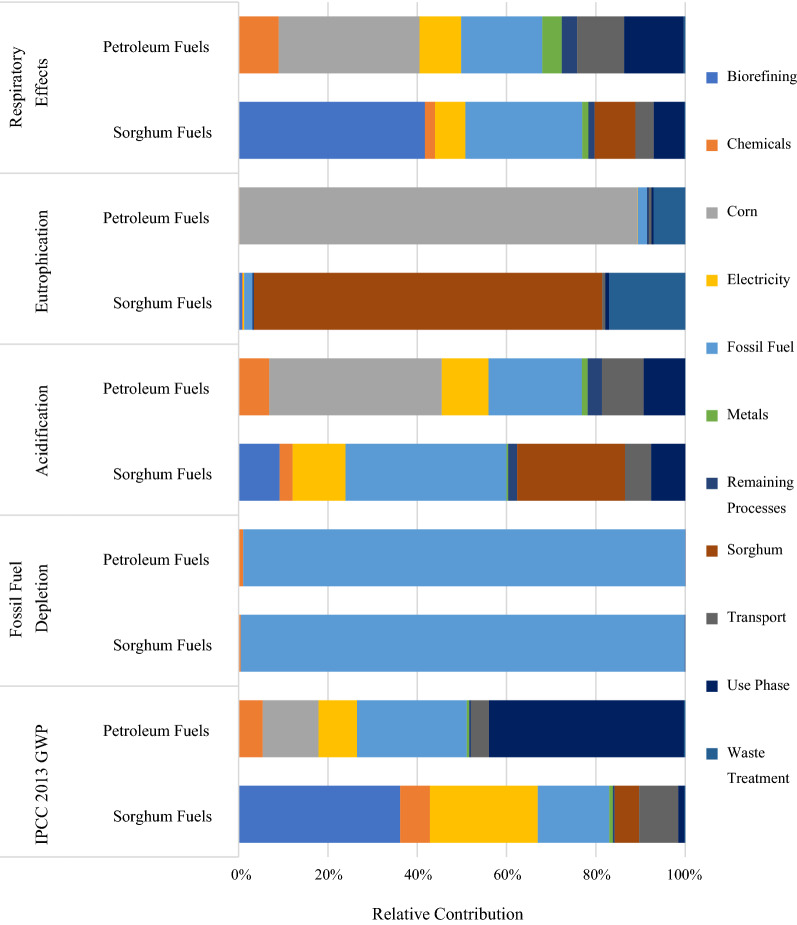
Process contribution analysis for impact categories. Left-to-right color order of each bar matches the top-to-bottom order of the legend

Compared to conventional fuels, sweet sorghum biofuels show a reduction of approximately 48% in GWP 100 a (reported in kg CO_2_ equivalent). Additionally, reductions were also found for ozone depletion (67%), fossil fuel depletion (52%), eutrophication (47%), and carcinogenic health impacts (15%). For smog, acidification, non-carcinogenic health impacts, and respiratory effects, petroleum fuels were found to have lower impacts of 21%, 16%, 55% and 49%, respectively, compared to sweet sorghum-derived biofuels. A detailed breakdown of processing step contributions to respiratory effects, eutrophication, acidification, fossil fuel depletion and GWP 100a are provided in Fig. 2 and the impacts where petroleum fuels had lower relative impacts that sorghum fuels with the exception of smog and non-carcinogenic impacts. The category ‘biorefining’ included in the process contribution results for all the sorghum biorefining steps directly after farming and transportation to the refinery. These processes include all material and energy requirements in the biorefinery: (1) juice extraction, (2) ethanol production, (3) bagasse drying, (4) bagasse particle size reduction, and (5) bagasse pyrolysis and upgrading.

Non-carcinogenic human health was not included in this LCIA, as it is dominated by heavy metal emissions and uptake which are incompletely modeled in the TRACI framework (heavy metals are taken up by plants, but the subsequent fate is not accounted for in the final fuel combustion step).

#### Climate change impact (GWP 100a)

The largest contributors to climate change, reported as GWP 100a, for sorghum fuels were found to be the biorefining, electricity, and fossil fuels. Biorefining processes that contributed most to greenhouse gas emissions included the hydrogen reforming step (57% of the burden) and the drying and pyrolysis operations (39%). The latter processes were large contributors to this impact category as a result of supplemental natural gas required for heat. Fossil fuels, primarily diesel used in farming equipment for sorghum and natural gas used in background processes also contributed to climate change.

The largest contributor to the total climate change impact for petroleum fuel production was found to be the fuel combustion step, or operation of the vehicle comprising 44%. By comparison, vehicle operation only contributed approximately 1% of the climate change impact for sorghum fuels. Other contributors for petroleum fuels included natural gas and corn farming (for production of E85), each contributing 25% and 13%, respectively.

#### Fossil fuel depletion

The process contributions for fossil fuel depletion are primarily the same as for climate change impact and are not further elaborated in this section.

#### Acidification

The largest contributors to acidification from sorghum biofuel production included fossil fuels (36%), sorghum farming (24%), and electricity (12% each). Fossil fuel consumption was dominated by natural gas utilization at the biorefinery—a combination of supplemental heat and feedstock for hydrogen production.

The largest contributors for petroleum-based fuels were corn farming (39%), process liquid fossil fuels (21%), and electricity (10%).

#### Eutrophication

Eutrophication impacts for sorghum- and petroleum-based fuel production were driven by sorghum farming (78%, 76 t) and corn farming (89%, 13 t), respectively. Closer inspection into the contributing factors for the farming steps showed nitrogen emissions to water dominated eutrophication impacts for both sorghum- and petroleum-based fuels. Eutrophication impacts were higher for corn because 198 kg nitrogen was required compared to 75 kg nitrogen required for sorghum to produce the reference flows of fuel to achieve the functional unit. These results seem atypical when considering that phosphorus emissions have higher eutrophication characterization factors compared to nitrogen. It is important to note that the TRACI impact assessment method combines both marine and freshwater eutrophication into one “Eutrophication” impact. Freshwater eutrophication is driven primarily by phosphorus emissions, while marine eutrophication is driven by nitrogen emissions. However, both sorghum and corn farming release more nitrogen to water than phosphorus, accounting for the reason for nitrogen dominating eutrophication impacts. With regard to the impact assessment comparison, the results show sorghum farming had lower eutrophication impacts compared with corn, yet the amount of sorghum produced exceeded that of corn produced for the petroleum-fuel blend by a factor of 5.8.

#### Respiratory effects

Three steps of biorefining, fossil fuels combusted for field work and background systems, and sorghum farming were the highest contributors to respiratory effects from sorghum biofuel production, each comprising 42%, 26%, and 9%, respectively.

Corn farming, processing fossil fuels and the use-phase were the highest contributors to respiratory effects from petroleum-based fuel production, each contributing 32%, 18%, and 13%, respectively.

#### Smog

Sorghum fuels had higher impacts in the category of smog compared to petroleum fuels. The largest contributors to smog from sorghum fuels were the biorefining processes (23%), fossil fuels (20%), transport (18%) and sorghum farming (12%). The largest contributors to smog in petroleum fuel production included emissions from transport (26%), corn farming (23%) and use-phase of vehicles (20%). Although the use phase is a smaller relative contribution to sorghum fuels, the numerical contribution was the same since the combustion of both fuel types has the same tailpipe emissions from the GREET model.

### Uncertainty results

The bootstrap Monte Carlo analysis of uncertainty described above showed that the functional unit for sweet sorghum biofuels had lower environmental impact than the petroleum fuels (*p* < 0.001) except for the carcinogens (*p* = 0.438) and ecotoxicity (*p* = 0.0245) categories. This is not entirely surprising as the range of characterization factors for these two categories is quite large and the driving factors associated with crop production are different between the two systems.

## Conclusions

There are important environmental benefits from sweet sorghum biofuels compared to conventional petroleum fuels. Sweet sorghum is a non-food crop in contrast to corn and sugarcane. The entire sorghum stalk is used in the integrated biorefinery, and the efficient capture of excess energy offsets the need to purchase to process heat and steam. Biochar used as fertilizer on the sorghum cultivation also resulted in lower fertilizer purchases, but this was a small effect. When comparing the environmental performance of the entire sorghum stalk to previous sorghum LCAs, the reduction of GWP (48%) falls well within the range of reported GHG reduction results. The functional unit of vehicle kilometers ensures a full accounting of impacts, including the use phase, ensuring a fair comparison of the fuel sources.

## Methods

### Goal and scope

The goal of this study was to determine the environmental feasibility of producing biofuel from all components of a sweet sorghum plant using a combination of biochemical and thermochemical (pyrolysis and hydrotreating) pathways and to compare these impacts to those of conventional petroleum-derived fuels. This quantification and comparison of environmental emissions and impacts can provide valuable information to decision-makers by quantifying the potential environmental benefits of hybrid processing in biomass-to-fuel pathways. An environmental profile of the entire production process also identifies hotspots within the product system that can guide future process improvements. Our scope includes a farm-to-wheel assessment of sorghum biofuels including: (1) stalk juice bioethanol, and (2) sorghum bagasse renewable gasoline and renewable diesel. This conceptual analysis includes all processes in the biofuel production cycle, starting with sorghum farming and terminating with biofuel utilization in a standard passenger automobile. The conversion processes are modeled as a small biorefinery to take advantage of heat integration.

### Functional unit

Previous LCA studies that focused on sorghum biofuels used a functional unit of volume or energy content of biofuels to assess and compare the environmental profiles of sorghum biofuel to petroleum fuels; however, this functional unit excludes the environmental credits of reduced use-phase emissions in biofuel combustion. For this reason, the functional unit used for this study was the sum of kilometers driven from the utilization of bioethanol, renewable gasoline, and renewable diesel resulting from one hectare of sorghum farming (76 t of biomass with 73 wt.% moisture). By choosing a reference of 1 ha of cropland, we can avoid the complication of allocation among the three types of biofuels produced from that hectare. For comparison to conventional fuels, we calculated reference flows necessary to deliver the same function of kilometers driven. The total distance that could be driven through end-use of all sorghum biofuels in a vehicle was estimated based on consumption of ethanol/gasoline blend (E85), renewable gasoline, and renewable diesel in internal combustion engine vehicles (ICEVs). The total kilometers that could be driven from the combustion of one hectare of all components of sweet sorghum-derived biofuel was found to be 89,990 km (Sect. "Sorghum biofuel yields").

### System boundary

**Fig. 3:**
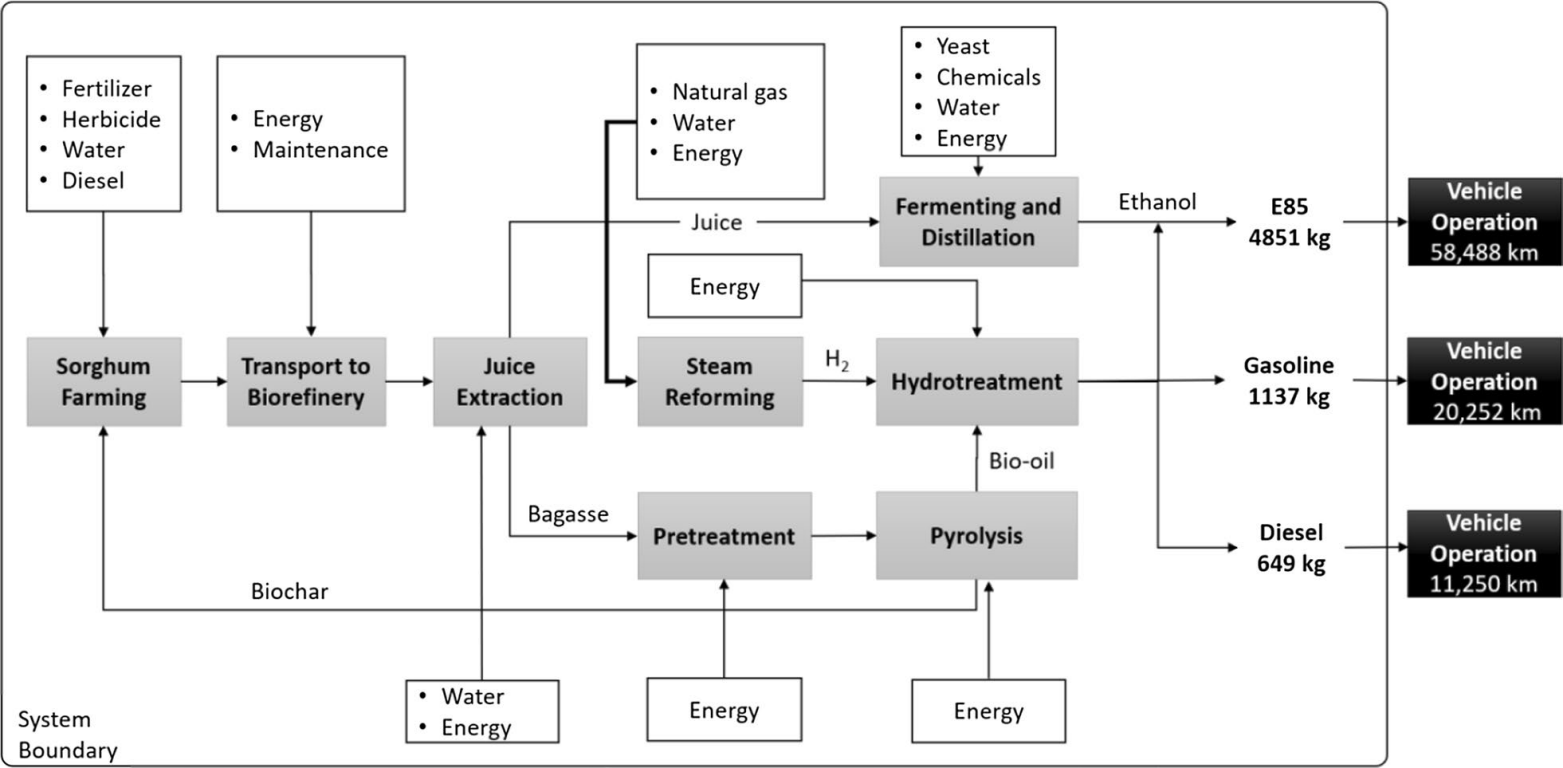
Sorghum biofuel production system boundary (adapted and ) modified from [29]

Our system boundary includes energy and material inputs for sorghum farming, stem juice extraction and fermentation, bagasse pretreatment and pyrolysis, hydrotreatment of bio-oil and blending of fuel, production of hydrogen gas for hydrotreatment, and ICEV operation and related emissions. A detailed system boundary figure is shown in Fig. 3.

A detailed description for each process included in this study is provided below. All assumptions made in the study for the life cycle inventory (LCI) are described for each process. The background LCI database used for this study was Ecoinvent 2.2 [20] and all models were built using SimaPro 9.1 [32].

### Life cycle inventory

#### Sweet sorghum farming

A ‘sweet sorghum farming’ system process from the Agri-footprint database was modified to represent the sorghum farming life cycle inventory (sorghum, at farm/US Mass). This process includes fertilizer, herbicide, irrigation water, and fuel, as well as emissions to air, water and soil. Transportation of fresh stalks from the farm gate to the biorefinery was also included, assuming a 50-km distance from the farm gate to the biorefinery. Input and output flows were modified for the reference flow of 76 ton of fresh stalks per hectare in this study. Fertilizer and herbicide use and diesel fuel for agricultural machinery were modified to match those of cultivating and harvesting 76 t based on results from Cai et al. [10]. The GREET model was used to update the emissions to air, water and soil from sorghum farming from the Agri-footprint unit process [38]. These figures were also scaled to model the basis of 1 ha of sweet sorghum farming. Fertilizer requirements were modified to account for application of biochar nutrients to the sorghum fields (Sect. "Bagasse pretreatment and pyrolysis").

#### Stem juice extraction and ethanol production

Manufacturer information for an industrial-scale roller mill was used to build the life cycle inventory for stem juice extraction. Energy required per kg of mass processed was estimated to be 10.85 kJ/kg of fresh sorghum stem (73 wt.% moisture) using a Demuth Standard Roller Mill Model 720 [15]. It was assumed that 87% of soluble sugars in the sorghum stalk were retained in the fluid following extraction [5]. Mass yields of grain, juice, and stalks obtained from Almodares and Hadi [5] were modified to match our sorghum biomass yield of 76 tons per hectare. Water was added in the juice extraction step to help with sugar retention, similar to sugarcane processing, in a mass ratio of 1:10 to the stem feed. The remaining 22.7 tons of bagasse were pretreated for pyrolysis, and residual grain (1.8 t) was retained as seed for the subsequent planting season.

An existing combined fermentation and distillation system process for sorghum stem was modified in order to reflect the characteristics of the stem juice produced in this study. This process included fermentation, distillation, and dehydration (final ethanol purity = 99.7%). Stalk juice was assumed to contain 14 wt.% sugar and have a potential to produce approximately 611 L of ethanol per ton of sugar [5]. Thus, an ethanol yield of 87 L per ton of stalk juice (14.6 kg stalk juice/kg of anhydrous ethanol) was used according to literature values [21]. An additional processing step was included following distillation utilizing a rectifying column and molecular sieve to produce a 99.7 wt.% ethanol solution as well as vinasse that was managed for leftover solids. The energy requirements used for these processes were based on an NREL process design for corn ethanol leftover solids [23]. Because the vinasse resulting from fermentation was primarily composed of water, this stream was classified as waste and treated using a wastewater process from Ecoinvent 2.2 [20].

#### Bagasse pretreatment and pyrolysis

Fast pyrolysis of feedstock requires small and dry particles of approximately 1 to 5 mm in diameter depending on the equipment used [34]. In addition, water that is released during pyrolysis can potentially provide a heat sink for energy, reducing the efficiency of the process to pyrolyze bagasse. Therefore, drying must be included in the pretreatment of bagasse to reduce moisture content. Energy inputs for a dryer were estimated with literature data for drying sawdust using two dryers in sequence: (1) a rotary wood dryer and (2) a hot oil heated Holoflight dryer (Lipuchina). The moisture content of wet bagasse was assumed to be 53.9 wt % after stalk juice extraction and entering wet bagasse was assumed to be at room temperature (25 °C) [5]. Electricity and energy requirements were determined for two dryers based on a mass flow rate of approximately 2 kg of bagasse per second with an exiting moisture content of 8 wt.% and 1 wt.% for the first and second dryers, respectively. Electricity requirements include a 100 hp dryer cyclone blower, a 50 hp fan, and a 20 hp motor.

In order to meet the recommended heat flux requirements for fast-pyrolysis (~ 600–1000 kJ/cm^2^), a comminution step to reduce particle size was also included in the analysis. Energy estimates for comminution were based on a knife mill with a ¼ inch screen size. Energy requirements were estimated at 28 kWh per ton of dry bagasse (1 wt.% moisture) for a mean particle size of 1.7 mm [7].

Yields for bio-oil, biochar, and syngas from pyrolysis were assumed to be consistent with theoretical NREL results of 75 wt.% bio-oil, 12 wt.% biochar, and 13 wt.% syngas, respectively [34]. Energy requirements for this process were also assumed to correlate with the NREL conservative value of 1000 kJ/kg of biomass. Residual syngas was combusted to provide a source of heat used in the drying step of the process. Biochar was recycled to the farming step where it was assumed to reduce fertilizer requirements by 0.91 kg ammonium nitrate per 100 kg of applied biochar based on nitrogen content of sorghum bagasse [11]. Other potential benefits of biochar to soil properties were not evaluated in this initial study.

#### Hydroprocessing of bio-oil and steam reforming

Our hydrotreatment process is a two-phase hydroprocessing method in which hydrogen and bio-oil are mixed with a solvent with high hydrogen solubility and comprise the feed stream. By introducing a solvent, hydrogen gas required to be circulated throughout the reactor is reduced as hydrogen dissolved in the feed stream is available to react [2]. This bio-oil/hydrogen/solvent feed stream enters a plug-flow reactor packed with a catalyst and hydroprocessing reactions including hydrotreating, hydrofinishing, hydrorefining and hydrocracking, take place. Products from hydroprocessing of bio-oil include gasoline, diesel, and syngas [3]. Yields of renewable gasoline, renewable diesel, and non-condensable gases were based on experimental data provided by Process Dynamics, Inc. Hydrogen gas requirements were also based on experimental requirements, amounting to 30.1 g of hydrogen gas per kg of bio-oil processed. Steam is a co-product generated from this process and was utilized as a heat source for the fermentation. Non-condensable gases produced during the hydrotreatment step were used as a source of heat for pyrolysis. Additional experimental data used for this unit process including process yields, electricity requirements, and steam heating values are listed in Additional file 1: Table S1. A steam reforming unit process was included to produce hydrogen gas for bio-oil hydrotreatment and subsequent fuel upgrading to transportation fuels. The inputs were stoichiometric amounts of natural gas and water (in gaseous form) that produced hydrogen gas and leftover steam. Natural gas required for steam was estimated at 164 MJ per kg of hydrogen produced. Residual steam was also produced in this process (approximately 12.3 MJ steam/kg hydrogen) and was utilized as an additional heat source for fermentation.

#### Vehicle operation with bioE85, renewable gasoline and renewable diesel

For bioethanol produced from stem juice, fuel blending was required in order to be used in standard passenger vehicles using an internal combustion engine. The two types of vehicles used for this analysis were spark ignition direct injection and compression ignition direct injection. Both types of vehicles are ICEVs. Due to the relatively larger quantity of ethanol produced compared to gasoline, a ‘bioE85’ blend was chosen to allow evaluation of the sorghum-based biofuels without supplemental fossil fuel blending. The bioE85 blend was assumed to comprise 85 vol% ethanol/15 vol% renewable gasoline using bioethanol produced from stem juice fermentation and sorghum renewable gasoline. The excess renewable gasoline produced (beyond that needed for the bioE85 blend) was used unblended in a standard passenger car. Although the market for pure renewable gasoline in the US is small, we assume it would not be blended with ethanol because it is already 100% renewable. Sorghum-derived renewable diesel was assumed to be used directly in diesel cars and have energy content equal to petrol-derived diesel. Fuel-blend energy content and vehicle energy requirements were derived from the GREET model and are available in Additional file 1: Table S2.

In order to provide a sound comparison of environmental impacts between sorghum fuels and petroleum fuels, the functional unit used for sorghum fuel production was also used for petroleum fuel production. Thus, a unit process for the total kilometers driven using fossil-derived gasoline–corn ethanol blend and diesel fuels was also modeled in the LCA software. Unit processes for the production of fossil-fuel derived gasoline, E85 with corn bioethanol, and fossil diesel, as well as operation of appropriate vehicles using these fuels were obtained from Ecoinvent 2.2 [20]. Since background fossil fuel extraction and combustion processes are included in our system boundary, it is important that some clarification is given for the contribution analysis results of sorghum- and petroleum-based fuels. Contributions to environmental impact categories from fossil fuels take two forms in this study: (1) non-use phase extraction and combustion, and (2) use-phase combustion. LCIA results therefore report a contribution analysis for each impact category in which contributions from non-use phase extraction and production processes are grouped as “Fossil Fuel” contributions and use-phase combustion processes are grouped as “Use Phase” contributions. The TRACI v2.2 LCIA framework linked with the life cycle inventory data described was used to calculate the midpoint impacts for the functional unit.

### Allocation and recycle streams

For the processing steps that generate co-products, mass allocation was utilized to distribute the environmental burden. However, it is important to note that the functional unit for this study incorporates the use of all end products (bioE85, renewable gasoline and diesel). Thus, this process does not require allocation of burden between the three biofuel products. Additionally, all other processes in the system boundary generated co-products that are simulated as being recycled in other unit processes, with co-products being forms of heat, biochar, and grain. Steam from the hydrotreating and steam reforming process was utilized as a heat source for the drying process. The on-site steam was not sufficient to complete the drying, thus supplemental heat from natural gas is also required. Non-condensable gases resulting from pyrolysis and hydrotreating steps were also utilized as a heat source for the ethanol distillation process. A summary of the energy and electricity inputs for the production of biofuels from one hectare of sweet sorghum and process heat utilization is provided in Additional file 1: Table S3.

We performed Monte Carlo (MC) simulations to evaluate the robustness of the conclusions of the comparison between the sweet sorghum-derived biofuels and fossil fuels. 1000 MC runs were performed in a pairwise fashion to ensure variation in shared background processes was not amplified in the results. We conducted a bootstrap statistical analysis to determine significance values for each impact category. Briefly, we selected, at random and with replacement, 100 pairs from the 1000 MC runs and performed a t-test. This was repeated 300 times to generate a distribution of p values, and we report significant differences when the upper 99% confidence limit of the distribution of p statistic values is less than 0.001 [31].

## Supplementary Information

**Additional file 1: Table S1.** Hydrotreatment process experimental data. **Table S2. **Vehicle and fuel data obtained from GREET (2020).

## Data Availability

The datasets during and/or analyzed during the current study are available from the corresponding author on reasonable request.
