# Supplementary material for: Life cycle impact assessment of biofuels derived from sweet sorghum in the U.S
Source: Biotechnol Biofuels. 2021 Aug 5;14:166. doi: 10.1186/s13068-021-02009-6 (PMC8340463; doi:10.1186/s13068-021-02009-6)
Supplement: Supplementary file 1 — Additional file 1: Table S1. Hydrotreatment process experimental data. Table S2. Vehicle and fuel data obtained from GREET (2020). [file 13068_2021_2009_MOESM1_ESM.docx]

**Table S1.** Hydrotreatment Process Experimental Data

|  | |
| --- | --- |
| *Process Yields* | 63:33:7 for steam, fuel, and non-condensable gases |
| *Fuel Yields* | 75:25 gasoline and diesel |
| *H_2_ requirements* | 30.1 g/kg bio-oil |
| *Electricity Requirements* | 6.93 MJ/kg bio-oil |
| *Non-condensable Gas Heating Value* | 16.6 MJ/kg |
| *Steam Heating Value* | 3.1 MJ/kg at 650 F |

**Table S2.** Vehicle and Fuel Data Obtained from GREET (2018)

| **Fuel** | **Energy Required (MJ/km)** | **Energy Content (MJ/m^3^)** | **Density (kg/m^3^)** | **Fuel Economy (kg/km)** |
| --- | --- | --- | --- | --- |
| **SIDI ICEV** | | | | |
| **E85** | 2453 | 23,125 | 782 | 0.0829 |
| **Renewable Gasoline** |  | 33,447 | 766 | 0.0561 |
| **Gasoline Blendstock** |  | 32,356 | 745 | 0.0565 |
| **CIDI ICEV** | | | | |
| **Low Sulfur Diesel** | 2350 | 36,234 | 847 | 0.0549 |
| **Renewable Diesel** |  | 36,234 | 889 | 0.0577 |

**Table S3**. Energy Use in Sorghum Biofuel Production (MJ/ha)

| **Production Step** | **Process Energy** | **Electricity^f^** | **Integrated Process Heat** |
| --- | --- | --- | --- |
| Farming | 7980^e^ | - | - |
| Juice Extraction | ^-^ | 741 | - |
| Fermentation/Distillation | 6381^c,d^ | 4839 | 4466^c,d^ |
| Dewatering | 404.1^g^ | 129 | - |
| Drying | 31962^a,g^ | 1207 | 14117^a^ |
| Comminution | - | 1057 | - |
| Pyrolysis | 10486^b,g^ | - | 9171^b^ |
| Hydroprocessing | - | 54763 | - |
| Steam Reforming | - | 3.9 | - |

^a^Heat from non-condensable gas from pyrolysis

^b^Heat from non-condensable gas from hydrotreating

^c^Heat from residual steam in hydrotreating

^d^Heat from residual steam from steam reforming

^e^Diesel and energy input for fertilizers and herbicides

^f^Medium voltage, at grid

^g^Natural gas combusted in industrial furnace
